# Development of a Cadaveric Shoulder Motion Simulator with Open-Loop Iterative Learning for Dynamic, Multiplanar Motion: A Preliminary Study

**DOI:** 10.3390/jcm12144596

**Published:** 2023-07-10

**Authors:** David Timothy Axford, Robert Potra, Richard Appleyard, Janos Tomka, Antonio Arenas-Miquelez, Desmond Bokor, Louis Ferreira, Sumit Raniga

**Affiliations:** 1 Department of Mechanical and Materials Engineering, Western University, London, ON N6A 3K7, Canada; 2 Faculty of Medicine, Health and Human Sciences, Macquarie University, Sydney, NSW 2109, Australia; 3 Department of Electrical and Computer Engineering, Western University, London, ON N6A 3K7, Canada

**Keywords:** active motion simulation, biomechanics, ex vivo, in vitro, reverse total shoulder arthroplasty, shoulder

## Abstract

Ex vivo shoulder motion simulators are commonly used to study shoulder biomechanics but are often limited to performing simple planar motions at quasi-static speeds using control architectures that do not allow muscles to be deactivated. The purpose of this study was to develop an open-loop tendon excursion controller with iterative learning and independent muscle control to simulate complex multiplanar motion at functional speeds and allow for muscle deactivation. The simulator performed abduction/adduction, faceted circumduction, and abduction/adduction (subscapularis deactivation) using a cadaveric shoulder with an implanted reverse total shoulder prosthesis. Kinematic tracking accuracy and repeatability were assessed using maximum absolute error (MAE), root mean square error (RMSE), and average standard deviation (ASD). During abduction/adduction and faceted circumduction, the RMSE did not exceed 0.3, 0.7, and 0.8 degrees for elevation, plane of elevation, and axial rotation, respectively. During abduction/adduction, the ASD did not exceed 0.2 degrees. Abduction/adduction (subscapularis deactivation) resulted in a loss of internal rotation, which could not be restored at low elevation angles. This study presents a novel control architecture, which can accurately simulate complex glenohumeral motion. This simulator will be used as a testing platform to examine the effect of shoulder pathology, treatment, and rehabilitation on joint biomechanics during functional shoulder movements.

## 1. Introduction

The glenohumeral joint of the shoulder has the largest range of motion of any joint in the body [1]. However, due to its lack of conformity, it can be unstable and is susceptible to injury and disease [2]. A tool that researchers use to better understand shoulder biomechanics is an ex vivo shoulder motion simulator, which allows researchers to simulate in vivo conditions in controlled environments to better understand shoulder pathology, treatment, and rehabilitation.

Ex vivo simulators can be divided into two main categories: static and dynamic simulators. Static simulators attempt to replicate in vivo joint loading for static shoulder positions [3] by pulling on the muscle tendons [3,4,5,6,7,8,9] or by applying external forces to the scapula and humerus [10,11,12,13]. Static simulators have been used to assess joint range of motion [14,15], the contribution of anatomical structures to joint stability [7,12,16], and joint contact pressures [5,9] at discrete joint positions. To assess biomechanical parameters across a continuous range of motion, dynamic simulators attempt to replicate in vivo joint motion in addition to joint loading. Early dynamic simulators simulated passive shoulder motion using external assistance provided by an investigator [17,18,19] or robotic manipulator [20]. The most advanced simulators do not require external assistance but can produce active muscle-driven motion by pulling on muscle tendons using computer-controlled actuators and software-based controllers [21,22,23,24,25,26,27,28].

To control joint motion, dynamic shoulder simulators use software-based controllers that control tendon loads, tendon excursions, or a combination thereof. The most common control method is tendon load control. An early implementation by Apreleva et al. applied equal, linearly increasing forces to all tendons to achieve arm elevation [29]. To produce more physiologic muscle load distributions, Wuelker et al. apportioned muscle loads based on muscle cross-sectional area [25], and Kedgley et al. incorporated muscle activation using electromyographic (EMG) data [19].

A limitation of these tendon load control designs is that they are not able to guide the shoulder along a desired trajectory because they do not incorporate kinematic feedback. To address this, Giles et al. implemented a closed-loop feedback control architecture, which adjusted the distribution of loads between muscles in real time based on measured kinematics to guide the shoulder along a desired path [21]. This controller grouped muscles into antagonistic pairs based on their primary function and adjusted the load distribution within each pair to drive the arm toward the target orientation. Although this closed-loop controller was able to achieve more accurate and repeatable shoulder movements than its predecessors, grouping muscles based on their primary function requires oversimplifying the muscle function and does not consider secondary and tertiary muscle functions [17,18]. It is also challenging to add or remove muscles without needing to redefine muscle groupings within the controller. Finally, closed-loop feedback architectures can be unstable and are often limited to performing simple planar motions at quasi-static speeds.

A control method, which is less common but has the potential to produce motions with higher repeatability and greater speeds, is tendon excursion control. This control method typically involves a ‘learning’ phase during which the arm is passively or actively articulated within a desired range of motion while the excursions of each tendon actuator are recorded [22,30,31]. These excursions are replayed to simulate active motion without external assistance. To achieve greater kinematic accuracy and more physiologic muscle forces, Sulkar et al. developed a hybrid tendon load and excursion controller, which used closed-loop feedback control [31]. This system was able to simulate subject-specific motion with a high level of accuracy, but like existing closed-loop tendon load controllers, the motions were limited to quasi-static speeds.

To overcome the simulation speed and motion profile complexity limitations of existing closed-loop control architectures, the aim of this study was to develop a control architecture capable of multiplanar motion with rotational speeds greater than 10 deg/sec. The second aim was to allow muscles to be removed or added without needing to alter the controller design by controlling each muscle independently instead of grouping muscles into antagonist pairs. This study presents a preliminary performance evaluation of the developed simulator and demonstrates its functionality for generating shoulder motions relevant to functional ranges.

## 2. Materials and Methods

### 2.1. Ex Vivo Shoulder Motion Simulator

**Figure 1:**
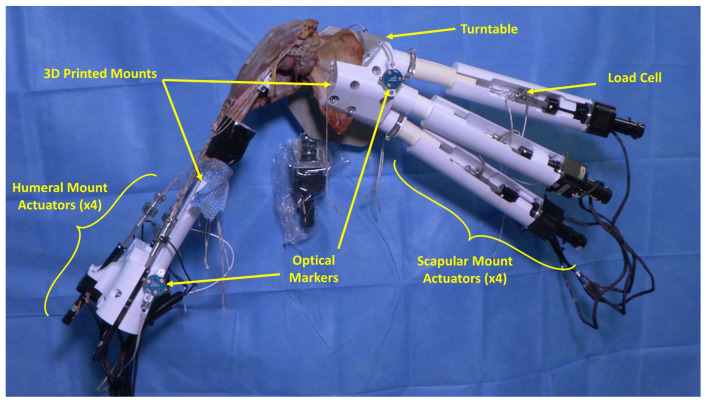
The developed ex vivo simulator used 3D-printed specimen-specific scapula and humeral mounts designed from the specimen CT scan. The two mounts included connection ports for linear actuators used to control the excursions of the infraspinatus, supraspinatus, subscapularis, teres minor (scapular mount actuators × 4; view of the infraspinatus actuator is obstructed by the subscapularis actuator), the three heads of the deltoid, and the short head of biceps (humeral mount actuators × 4). A uniaxial load cell was placed at the end of each actuator to measure tendon force. The scapula was mounted onto a single degree of freedom turntable to simulate scapular rotation. Optical markers were mounted onto both the humerus and scapula mounts to measure joint kinematics.

An ex vivo shoulder motion simulator was developed to simulate active glenohumeral joint motion by actuating eight shoulder muscles (Figure 1). Prior to specimen dissection and testing, a single cadaveric specimen (69 yr, female, full arm, including scapula, humerus, and clavicle) was CT scanned, and a 3D model of the bony anatomy was created by manually segmenting the bones from the CT (Mimics, Materialise NV, Leuven, Belgium). Using the 3D model, specimen-specific scapular and humeral mounts were designed and 3D printed (SOLIDWORKS, Dassault Systèmes, Vélizy-Villacoublay, France). These mounts were used to fix the specimen to the simulator test frame and contained connection ports for linear actuators. The actuators were used to control the excursions of the three heads of the deltoid (anterior, middle, and posterior), the four rotator cuff muscles (subscapularis, supraspinatus, infraspinatus, and teres minor), and the short head of biceps. Each connection port was optimally placed based on the specimen CT scan to ensure that each actuator had a direct line-of-pull on its corresponding tendon. The lines-of-pull were determined using the origin and insertion of each muscle. For cases where a muscle had a broad attachment site (subscapularis, infraspinatus, and deltoid scapular attachments), the actuator was oriented, so that its line-of-pull would pass through the midpoint of the attachment site. The scapular mount contained connection ports for the four rotator cuff muscle actuators (size 23, Picard Industries, Albion, NY, USA), and the humeral mount contained connection ports for the three deltoid actuators and the short head of biceps actuator (size 17, Picard Industries, Albion, NY, USA). To attach the humeral mount onto the distal humerus, a 3D-printed specimen-specific cutting guide was used to transect the humerus distal to the deltoid insertion. A uniaxial load cell (Honeywell Model 31 Series, Honeywell International Inc., Charlotte, NC, USA) was placed at the end of each actuator to measure tendon force. The scapula was mounted onto an actuator-driven turntable (size 23, Picard Industries), which maintained a 2:1 ratio between glenohumeral elevation and scapulothoracic upward rotation. Optical markers were mounted onto the scapular and humeral mounts to track joint kinematics (Optotrak Certus, Northern Digital Inc., Waterloo, ON, Canada).

### 2.2. Motion Control

To control joint motion, the simulator used an open-loop tendon excursion controller with iterative learning. The controller produced a desired motion by prescribing actuator excursions calculated from specimen-specific tendon excursion maps. These tendon excursion maps were created using a shoulder calibration procedure. During this procedure, the arm was passively articulated through its range of motion while each actuator applied a constant force of 20 N to its corresponding tendon. The measured joint angles and actuator excursions were recorded and were used to map the excursion of each actuator as a function of joint angle. During active motion, the prescribed excursions were played in an open-loop fashion, and after the simulated motion was completed, the controller measured the kinematic error between the desired motion path and the actual path achieved. This error was used to adjust the actuator excursions using the tendon excursion maps to reduce kinematic errors for subsequent iterations of the same motion. This process of adjusting the actuator excursions based on measured kinematic data was called ‘kinematic error compensation’ and was performed iteratively for each motion until the kinematic errors were sufficiently small.

### 2.3. Specimen Preparation

The cadaveric specimen used in this study was stored at −20 °C prior to testing and was slowly thawed over 4–5 days in a refrigerator (4–6 °C) to minimize tissue damage [32]. On the day prior to testing, the specimen was removed from the refrigerator and brought to room temperature for specimen dissection.

The specimen was dissected to isolate the eight muscles being actuated. The origin of the deltoid muscle was left intact on the acromion and clavicle, and the three heads were isolated and detached from their insertions on the humerus. In this way, native muscle wrapping was maintained over the shoulder, and no actuating cables crossed the shoulder joint. The origin of the short head of the biceps tendon was left intact on the tip of the coracoid, and the muscle belly and long head of biceps tendon were removed. The rotator cuff muscle origins were separated from the scapula, and the muscle bellies were removed. All other soft tissues were removed from the scapula, humerus, and sternal half of the clavicle (medial to the anterior deltoid origin). The clavicle was stabilized by fixing it to the coracoid with a 2.5 mm screw using the Bosworth screw fixation technique.

**Figure 2:**
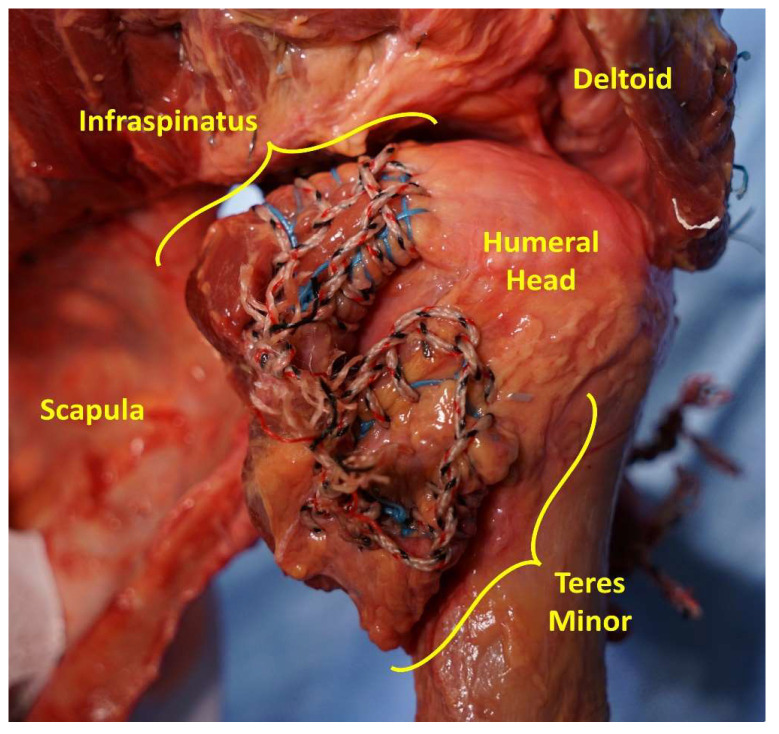
This figure shows the suturing technique used to connect each tendon to the corresponding actuator. Sutures were inserted into each muscle tendon in two layers. The first layer was a Krackow stitch using either number 2 OROTHOCORD™ or DYNACORD™ braided composite suture (DYNACORD™ shown here in blue). The second layer was a Krackow stitch using braided Dacron fishing line (red, white, and black suture). This figure shows the suture for the infraspinatus and teres minor and is representative of all muscle sutures.

Sutures were inserted into each muscle tendon and tied to the corresponding actuator. The sutures were inserted in two layers (Figure 2). The first layer was a running locking stitch (Krackow stitch) with a number 2 ORTHOCORD™ or DYNACORD™ braided composite suture (DePuy Synthes, Raynham, MA, USA). This suture created a firm base of tissue for the second suture. The second layer was a running locking stitch (Krackow stitch) with a braided Dacron fishing line (200 lb Tuf-Line Braided Dacron, O. Mustad & Son, Gjøvik, Norway), which was sutured around the first suture layer. Both layers were sutured along the outer edges of the distal part of the muscle/tendon.

**Figure 3:**
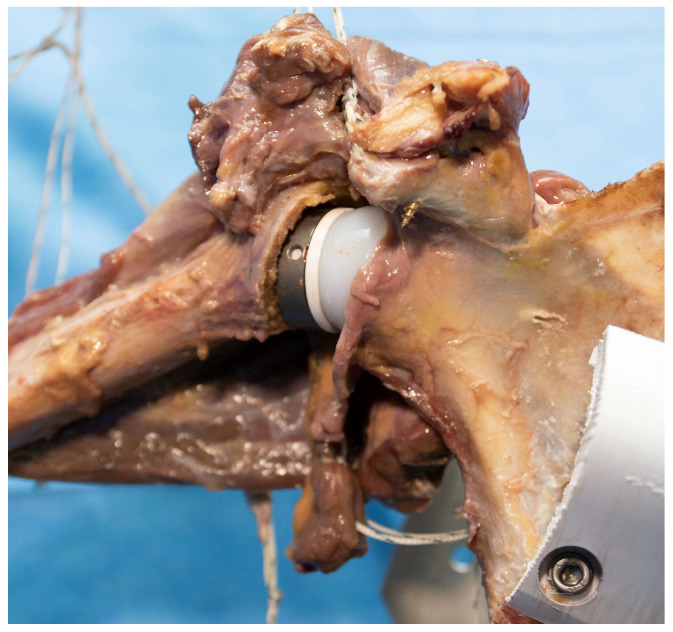
A custom reverse total shoulder arthroplasty prosthesis was implanted into the shoulder. This prosthesis was designed based on the Mathys Affinis Fracture Inverse system.

Once the specimen was prepared, it was returned to the refrigerator until testing began the following morning. On the day of testing, the specimen was mounted on the simulator using the scapular and humeral mounts. The MotionMonitor xGen (Version 3.55.7.0, Innovative Sports Training, Inc., Chicago, IL, USA) software was used to digitize bony landmarks and construct anatomical coordinate systems for the humerus and scapula according to International Society of Biomechanics (ISB) standards [33]. Finally, a custom reverse total shoulder arthroplasty (RTSA) prosthesis was implanted into the shoulder (Figure 3). This implant was a Grammont style prosthesis based on the Mathys Affinis Fracture Inverse system (Mathys Medical, Bettlach, Switzerland).

### 2.4. Simulated Shoulder Movements

#### 2.4.1. Abduction/Adduction

The first motion simulated was abduction/adduction with a peak glenohumeral rotation speed of 25 degrees/second. Glenohumeral elevation varied between 15 and 60°; the plane of elevation was held constant at 70° (20° anterior to the scapular plane); and axial rotation was held constant at −30° (30° of external rotation). This motion was repeated for three cycles, and the joint kinematics and muscle forces were averaged across all three cycles.

#### 2.4.2. Faceted Circumduction

The second motion simulated was a modified circumduction motion called faceted circumduction. The motion was modified by cutting the circumduction ellipse with a cord to create discontinuities in the motion path, which were intended to challenge the controller. Glenohumeral elevation varied between 15 and 35°; the plane of elevation varied between 50 and 90° (angles less than 90° are anterior to the scapular plane); and axial rotation was held constant at −30°. Five circumduction cycles were performed with the facet placed in different locations around the ellipse for every motion cycle. Moving the facet around the circumduction ellipse was intended to further challenge the controller with a non-repetitive motion. Each cycle had a duration of 10 s, which corresponded to about 10 degrees/second of glenohumeral rotation.

#### 2.4.3. Abduction/Abduction—Subscapularis Deactivation

For the final motion, abduction/adduction was performed with glenohumeral elevation from 15 to 35°; the plane of elevation was constant at 70°; and axial rotation was constant at −30°. The motion was initially performed with all eight muscles actuated. The subscapularis actuator was then deactivated, keeping the linear actuator in the fully extending position, such that there was no tension applied to the subscapularis tendon. With the deactivated subscapularis, the same actuator excursions were replayed for the remaining seven muscles, and changes in the kinematics and muscle forces were observed. Finally, the kinematic error compensation routine was used to compensate for the missing subscapularis muscle by adjusting the excursions of the remaining seven muscles in an attempt to restore joint kinematics. At each stage, two motion cycles were performed, and the resulting joint kinematics and muscle forces were averaged across cycles.

### 2.5. Performance Outcome Metrics

For each motion, kinematic tracking accuracy was assessed by calculating the maximum absolute error (MAE) and root mean square error (RMSE) between the target motion path and the actual path achieved. The simulator’s repeatability was assessed by calculating the average standard deviation (ASD) in joint angles and muscle forces across three repeated trials of abduction/adduction.

## 3. Results

### 3.1. Abduction/Adduction

**Figure 4:**
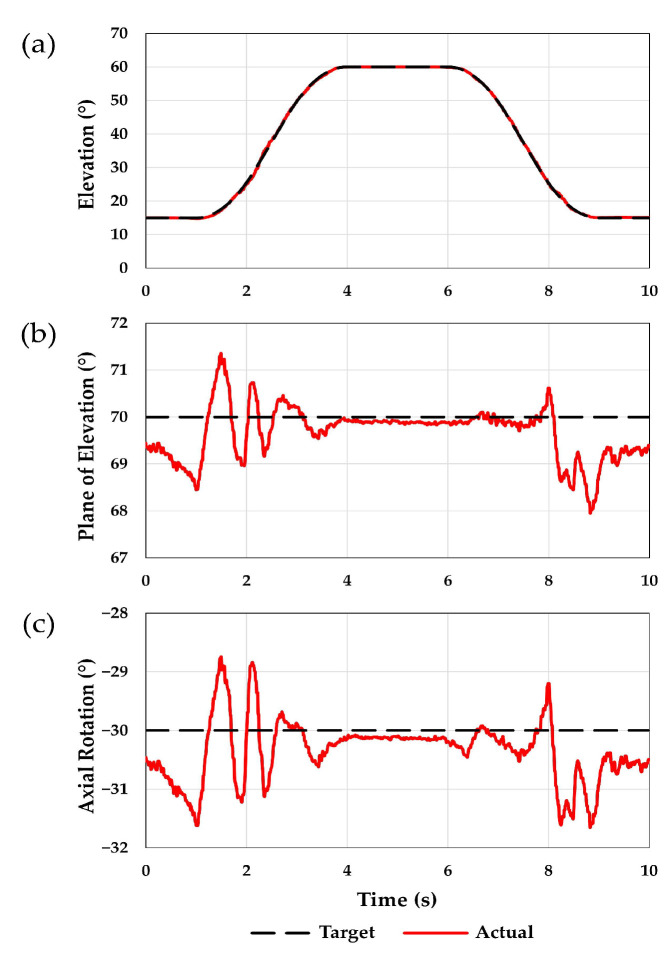
The simulator was used to generate an abduction/adduction motion at 25 deg/s. Three motion cycles were performed, and the average of the three cycles was calculated. This figure shows a comparison between the target joint angles (black) and the actual angles (red) achieved for (**a**) elevation, (**b**) plane of elevation, and (**c**) axial rotation. Plane of elevation angles less than 90° are anterior to the scapular plane, and negative axial rotation angles indicate external rotation.

**Figure 5:**
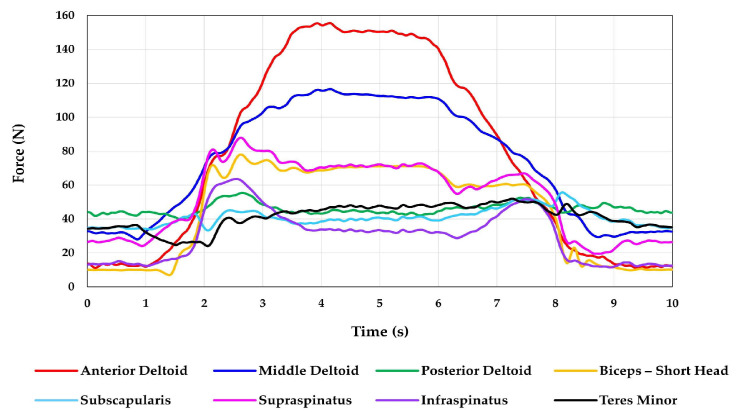
This figure shows the resulting muscle forces for an abduction/adduction motion. Three motion cycles were performed, and the average forces for the three motions were calculated.

The MAE observed for abduction/adduction was 1.8, 2.2, and 2.1° for elevation, plane of elevation, and axial rotation, respectively. The RMSE was 0.3, 0.7, and 0.7° for elevation, plane of elevation, and axial rotation, respectively. The ASD in joint angles between the three motion cycles was 0.2° for all three joint angles. The ASD in muscle forces between the three motion cycles were 1.4, 1.2, 1.2, 0.6, 1.9, 1.5, 1.2, and 2.7 N for the anterior deltoid, middle deltoid, posterior deltoid, short head of biceps, subscapularis, supraspinatus, infraspinatus, and teres minor, respectively. A comparison between the target joint angles and the actual joint angles achieved is shown in Figure 4. The resulting muscle forces are shown in Figure 5.

### 3.2. Faceted Circumduction

**Figure 6:**
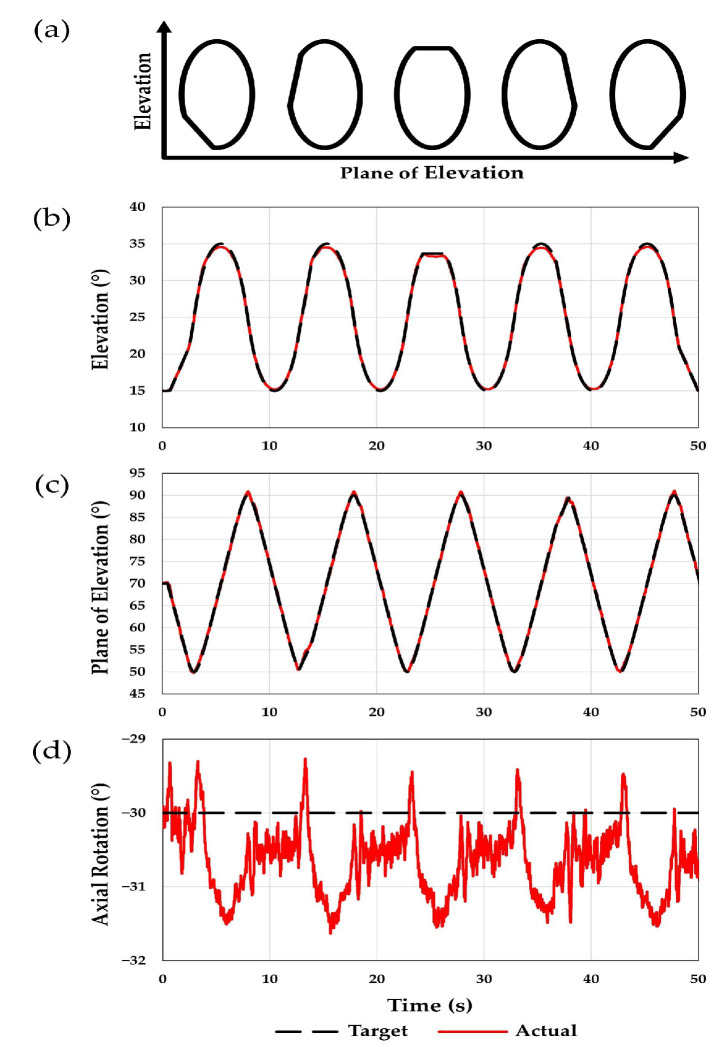
The simulator was used to perform a faceted circumduction motion. (**a**) Five cycles of the motion were performed, and the location of the facet changed along the circumduction ellipse for each motion cycle. This is a comparison between the target joint angles (black) and the actual angles (red) achieved for (**b**) elevation, (**c**) plane of elevation, and (**d**) axial rotation. Plane of elevation angles less than 90° are anterior to the scapular plane, and negative axial rotation angles indicate external rotation.

**Figure 7:**
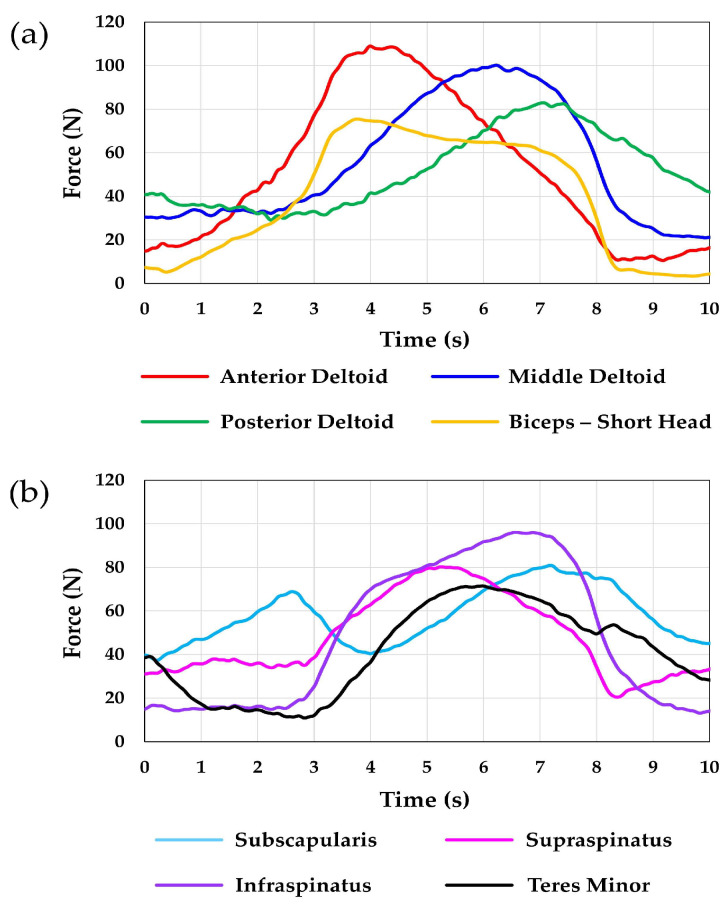
This figure shows the resulting muscle forces for a single faceted circumduction motion cycle (first cycle). The eight muscles were separated into two groups of four muscles for clarity: (**a**) muscles actuated from the humeral mount and (**b**) muscles actuated from the scapular mount.

The MAE observed for faceted circumduction was 0.6, 1.4, and 1.6° for elevation, plane of elevation, and axial rotation, respectively. The RMSE was 0.2, 0.3, and 0.8° for elevation, plane of elevation, and axial rotation, respectively. Figure 6 shows a comparison between the target and the actual joint angles achieved. The resulting muscle forces for a single cycle are shown in Figure 7. For this motion, the arm began at low elevation with the arm slightly anterior to the scapular plane. As the arm elevated, it began moving more anteriorly and then eventually moved posteriorly into the scapular plane as the arm was adducted. Figure 7b shows that at the beginning of each circumduction cycle, the anterior deltoid had the greatest force. Then, as the arm began to move posteriorly toward the scapular plane, the middle deltoid force exceeded the anterior deltoid. Finally, toward the end of each cycle, the posterior deltoid force increased as the middle and anterior deltoid forces decreased.

### 3.3. Abduction/Adduction—Subscapularis Deactivation

**Figure 8:**
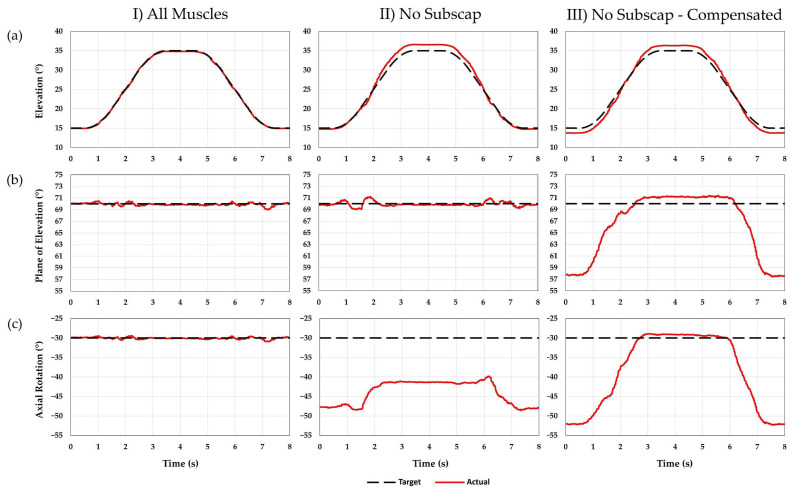
Two cycles of an abduction/adduction motion were performed, and the average of the two cycles was calculated. This is a comparison between the target joint angles (black) and the actual angles (red) achieved for (**a**) elevation, (**b**) plane of elevation, and (**c**) axial rotation. Plane of elevation angles less than 90 degrees are anterior to the scapular plane, and negative axial rotation angles indicate external rotation. (**I**) An initial motion was performed by actuating eight muscles in the shoulder. (**II**) The subscapularis was deactivated, and the actuator excursions for the remaining muscles were replayed. (**III**) Kinematic error compensation was used to compensate for the missing subscapularis.

**Figure 9:**
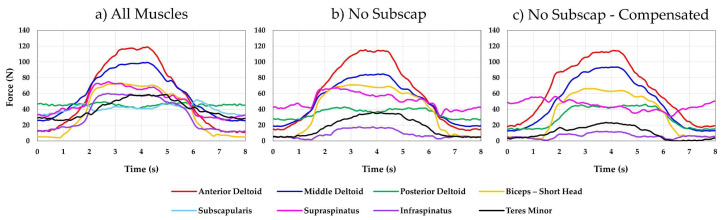
This figure shows the resulting muscle force for abduction/adduction with (**a**) eight muscles in the shoulder being actuated, (**b**) the subscapularis deactivated, and (**c**) compensating for the missing subscapularis using kinematic error compensation.

The MAE observed while all eight muscles were activated was 0.6, 1.0, and 1.0° for elevation, plane of elevation, and axial rotation, respectively. The RMSE was 0.2, 0.3, and 0.3° for elevation, plane of elevation, and axial rotation, respectively. Replaying the same actuator excursions with the subscapularis deactivated resulted in an MAE of 2.0, 1.3, and 18.9° for elevation, plane of elevation, and axial rotation, respectively. The RMSE was 1.1, 0.4, and 14.2° for elevation, plane of elevation, and axial rotation, respectively. Finally, using kinematic error compensation to restore joint kinematics without the subscapularis muscle resulted in an MAE of 2.0, 12.7, and 22.4° for elevation, plane of elevation, and axial rotation, respectively. The RMSE was 1.1, 6.5, and 12.8° for elevation, plane of elevation, and axial rotation, respectively. Figure 8 shows a comparison between the target and the actual joint angles achieved for all three conditions. Figure 9 shows the resulting muscle forces.

## 4. Discussion

Despite what their name suggests, existing dynamic ex vivo shoulder simulators do not simulate the true dynamic motion of the shoulder. Instead, due to the limitations of existing controller designs, the motions are limited to quasi-static speeds. This allows investigators to ignore inertial effects when simulating motion but prohibits studying shoulder biomechanics in the context of dynamic motion. Many of the functional movements we perform during daily activities are not quasi-static, and therefore, inertial effects should not be ignored. The first step toward conducting dynamic shoulder motion studies is to develop a control architecture, which can simulate dynamic motions. Using a closed-loop feedback control architecture, Giles et al. achieved a joint motion tracking RMSE of less than 1 degree for all three joint angles with a glenohumeral rotational speed of approximately 5 degrees/second [21]. The controller presented here showed a high level of accuracy and repeatability when simulating abduction/adduction with a glenohumeral rotational speed of 25 degrees/second. At this rotational speed, the RMSE for all three joint angles was less than 0.7°, and the ASD across three motion cycles was less than 0.2° for all three joint angles and less than 2.7 N for all muscle forces.

Not only do many of the shoulder movements associated with daily tasks involve inertial effects, but their trajectory can be complex. However, most of the motions that are simulated using existing simulators are simple planar motions, which only vary a single rotational degree of freedom while holding other degrees of freedom constant. Recently, Sulkar et al. simulated multi-planar, subject-specific scapula and glenohumeral kinematics using a cadaveric shoulder simulator with glenohumeral rotation speeds below 1 degree/second [31]. Sulkar reported mean absolute errors of less than 3° for glenohumeral elevation, plane of elevation, and axial rotation. In this study, multi-planar motion was simulated by performing faceted circumduction with a glenohumeral rotation speed of around 10 degrees/second. The resulting RMSE for all three joint angles was less than 0.8°, demonstrating high accuracy when simulating complex, multi-planar motions at increased speeds.

One challenge with using a tendon excursion controller instead of a tendon load controller is that physiologic forces can be difficult to achieve because the forces are not being directly prescribed but are an outcome metric of the simulated motion. As demonstrated in this study, even though the forces were not prescribed, the force relationships observed between muscles were consistent with what would be expected in vivo. This was demonstrated most clearly by observing the transfer of load between the three heads of the deltoid during faceted circumduction.

Many existing control architectures group muscles based on their primary function. This presents two challenges. The first is that this oversimplifies the muscle function. Ackland et al. have shown that an individual muscle can affect more than one rotational degree of freedom, and how it affects joint motion can change with arm orientation [17,18,34,35]. The controller presented here captured the multidimensional function of each muscle by mapping its excursion as a function of joint orientation during shoulder calibration. As a result, the controller did not assume the function of any muscle but learned the function of each muscle through the measured tendon excursions. The second challenge that organizing muscles into antagonistic groups presents is that muscles cannot be added to or removed from the controller without needing to alter the controller design. Being able to add or remove muscles is critical when studying certain aspects of shoulder biomechanics, such as whether to repair the subscapularis tendon following RTSA implantation. Some suggest that there are no clinical benefits to repairing the tendon [36], while others recommend repairing the subscapularis whenever possible [37].

In this study, the effect of deactivating the subscapularis was observed for glenohumeral abduction/adduction with an implanted RTSA prosthesis. Immediately after the subscapularis was deactivated, there was a loss of internal rotation, which was expected, given the primary function of the subscapularis is internal rotation of the humerus. There was also a small increase in the elevation angle and a small decrease in the anterior and middle deltoid forces. This suggests that the subscapularis was secondarily acting as an adductor of the shoulder [38,39]. With the implantation of a Grammont style prosthesis, the humerus was distalized, and the insertion of the subscapularis tendon was moved inferior to the joint center of rotation, resulting in the subscapularis tendon contributing to adduction of the shoulder. Finally, when the excursions of the remaining muscles were adjusted to compensate for the missing subscapularis, internal rotation was not restored at low angles of elevation, which is an observation often made clinically [40], and the humerus moved anteriorly away from the scapular plane. At low angles of elevation, the kinematic error compensation routine caused an increase in the anterior deltoid force and a decrease in the posterior deltoid force. Without the presence of a dominant internal rotator of the humerus, the controller attempted to use the anterior and posterior deltoid to restore axial rotation. However, since the anterior and posterior deltoid significantly affect the plane of elevation of the humerus, the deltoid was not able to compensate for the loss of internal rotation while maintaining the proper plane of elevation.

Another limitation of the current simulator technology is that the three heads of the deltoid are commonly replaced with cables, which are secured at one end to the deltoid tuberosity and at the other end to actuators mounted to the scapula. Using cables in place of the muscle belly does not properly distribute the deltoid wrapping force across the humeral head and results in stresses through the acromion that are not consistent with what would be expected in vivo. The simulator presented here did not replace the deltoid muscle with cables but left it intact and actuated the three heads from the humeral side. This maintained proper deltoid wrapping and gave a more physiologic force distribution along the acromion and clavicle.

This study had a number of limitations. Firstly, this is a preliminary performance assessment, which used a single cadaveric specimen. A comprehensive performance assessment needs to be performed with a larger sample size to further support the performance metrics presented in this study. Secondly, scapular rotation was controlled with a fixed ratio in a single degree of freedom. Previous studies have shown that three-dimensional rotation of the scapula substantially contributes to arm elevation and that these rotations do not occur in fixed ratios [41]. To address this, future development will focus on incorporating full six degree of freedom motion of the scapula. Finally, although the motion speeds produced in this study approach the dynamic range, the mass and mass moment of inertia of the humeral mount and actuators were not tailored to simulate the native arm. Although this was not critical to conducting a performance evaluation, future clinical studies involving inertial effects will need to ensure that the mass and mass moment of inertia of the distal arm are considered.

## 5. Conclusions

In conclusion, this early evaluation of a novel control architecture for shoulder motion simulation showed that an open-loop tendon excursion controller with iterative learning can produce fast and complex motions with a high level of accuracy and repeatability. This control architecture will be further tested by conducting a comprehensive performance assessment to validate this control technique. Overall, this initial performance assessment demonstrated that this control technique has the potential to be used to study dynamic shoulder movements associated with daily living tasks to better understand shoulder pathology, treatment, and rehabilitation.

## Data Availability

The data presented in this study are available on request from the corresponding author. The data are not publicly available due to privacy and ethical concerns.
